# Editorial: Glycoconjugate antigen processing and immune response

**DOI:** 10.3389/fmolb.2023.1350141

**Published:** 2023-12-18

**Authors:** John F. Cipollo, Giuseppe Stefanetti

**Affiliations:** ^1^ United States Food and Drug Administration, Silver Spring, MD, United States; ^2^ University of Urbino Carlo Bo, Urbino, Marche, Italy

**Keywords:** organ transplant, vaccine processing, glycoprotein antigen, innate immunity, adaptive immunity, glycoconjugate vaccines

This Research Topic aims to advance our comprehension of glycoconjugate interactions within the host immune system. These diverse molecules, ubiquitously present in nature, exhibit structural variability and function as both antigens for processing and signaling agents to engage the host’s immune system. The contributions within this Research Topic delve into various aspects, including inflammatory diseases, elements related to tissue and organ transplantation, glycoconjugate vaccines and antigen processing, and the interactions between viral glycoprotein antigens and innate immune factors.

The human immune system exists as a highly complex interplay between adaptive and innate immune responses in equilibrium between immunity and inflammation. Glycoconjugates of viral envelopes, bacterial surfaces, aberrant host forms, and cancer cell surfaces can serve as potent antigens and other immune stimuli, giving rise to a range of downstream immune processes that remain incompletely understood. For example, while the ABO blood group antigen system was discovered by Karl Landsteiner well over 100 years ago, the immune tolerance of these antigens as an adaptive phenomenon in tissue and organ transplant remains an active area of research. Viral antigens often possess heavily glycosylated antigenic proteins on their surfaces and interact with components of both the innate and adaptive immune system. Viral pathogens can mask antigenic surfaces by introduction of new glycosylation sites, removing them or shifting their positions as they pass through the host populations, thus changing antigenic sites and regions of the protein that interact with the immune system. Many pathogenic bacteria produce capsular polysaccharides during the invasive stage of disease as part of an evasion strategy. These molecules have been successfully utilized in vaccines. As vaccine antigens, they generate T-cell-independent short-lived antibody responses that are not protective in young infants. However, the conjugation of these polysaccharides to a protein carrier was a breakthrough in vaccine technology in the late 1980s, allowing the generation of immunogens associated with T-cell help, long-lasting B-cell memory, and protection in young infants. Glycoconjugates are also involved in aberrant cytotoxic/degenerative diseases such as Crohn’s disease. The articles in this Special Topic delve into these intricate areas, shedding light on the multifaceted involvement of glycoconjugates in immune responses, infectious diseases, and immune-related disorders.

Uri Galili review explores the importance of alpha-galactosyl (alpha-gal) epitopes in ABO blood type allografts, playing a critical role in initiating tissue-rejection mechanisms. It highlights the absence of alpha-galactosyltransferase in B blood types, resulting in the production of antibodies against alpha-gal that contribute to acute rejection in mismatched allografts—a significant concern in transplantation. The work delves into methods aimed at eliminating anti-B antibodies and modifying mononuclear cells using alpha-galactosyltransferase with the goal of inducing tolerance in the host immune system. These strategies are aligned with the manuscript’s emphasis on immunomodulation for successful transplants. Moreover, the review widens the discussion on alpha-gal as a potent chemotaxant, presenting its potential applications in enhancing vaccines, chemotherapy, and reversing myocardial damage. This multifaceted perspective underscores alpha-gal epitopes’ versatility beyond transplantation, indicating their relevance across various medical fields.

Micoli et al. offer an extensive compilation diving into the development, efficacy, and immunogenicity of vaccines using glycoconjugate technology. It encompasses an array of bacterial pathogens such as *Salmonella*, *Shigella*, *Haemophilus influenzae* type b, and *Streptococcus pneumoniae*, among others. Their discussions span carrier proteins, conjugation chemistry, polysaccharide size, and structural impacts, also exploring various adjuvants’ influence on immune responses. The report specifically explores the structural aspects, production methods, and vaccine effectiveness of polysaccharide conjugate vaccines. Their research highlights critical factors—polysaccharide length, protein–saccharide ratio, and the choice of conjugate protein—vital in optimizing immune response and antigen processing for enhanced vaccine efficacy. This comprehensive overview encompasses advancements, challenges, and methodologies within glycoconjugate vaccines, offering insights into combating diverse bacterial infections effectively.

Still in the field of glycoconjugate vaccines, Di Benedetto et al. investigate shigellosis, a major cause of diarrhea in young children in lower-income countries, and the urgent need for effective vaccines due to rising antimicrobial resistance. A four-component vaccine candidate utilizing Generalized Modules for Membrane Antigens (GMMA) technology—a method involving the extraction of outer membrane vesicles from bacteria—is being tested to protect against various *Shigella* strains. Comparing this vaccine with traditional glycoconjugates in mice and rabbits, the GMMA-based vaccine shows promise, inducing stronger and broader immune responses. However, the differences observed in animal models emphasize the importance of understanding how these findings relate to human responses as these vaccines move through clinical trials.

Parsons et al. work delves into the zoonotic potential of an H4 influenza lineage in southern China, exploring mutations within the virus’s hemagglutinin receptor binding site. These mutations allow the virus to adapt to sialyl-α2,6 receptors present in swine and humans. Their study investigates the role of SP-D, a protein binding high mannose glycans, in facilitating the removal of pathogens such as influenza. Their findings suggest that the H4 virus in this lineage exhibits susceptibility, potentially posing a lower threat to humans compared to highly pathogenic avian influenza strains such as H5 and H7.

Hasler et al. investigate the impact of bacterial glycosyl hydrolases in the gut and their impact on gut health. These enzymes form a crucial part of the bacterial strategy for scavenging carbohydrate sources from gastric surfaces, aiding in their utilization as a carbon source. However, this process can inadvertently damage the glycoproteins involved in critical cellular adhesion and activation processes within the gut lumen, a phenomenon observed in conditions such as colitis. Their investigation encompasses various glycosyl hydrolases, particularly those targeting fucosyl, galactosyl, and sialyl linkages, unveiling how specific hydrolases impact different tissues and shedding light on the pathological effects associated with their actions.

Krylov et al. examine α-mannan chains’ structural impact on their antigenicity, specifically focusing on the anti-Saccharomyces cerevisiae antibodies (ASCA) epitope. Using synthetic oligosaccharides mimicking fungal mannan fragments, they explore how structural alterations affect their recognition by human serum antibodies. These findings suggest that elongating the ASCA epitope with additional mannose residues enhances antibody recognition, potentially indicating higher immunogenicity. This research paves the way for improving ELISA test systems and developing more effective fungal-based diagnostic kits by transitioning to synthetic mimetics with well-defined structures.

This special topic delves into various facets of mammalian immunity influenced by glycoconjugates, offering valuable insights into therapeutic strategies. The articles explore avenues for enhanced vaccine design, emphasizing cytotaxis targeting, antigen structure refinement, and immune cell activation against cancers. As our comprehension of glycoconjugate interactions with the immune system deepens, the way toward a more comprehensive understanding of vaccination, autoimmune conditions, inflammatory mechanisms, and their modulation is realized. This series represents a small but significant contribution toward advancing this field of study.

